# Brief intervention for stress management and change in illness perception among hypertensive and normotensive workers: pilot study and protocol

**DOI:** 10.1186/s41155-017-0080-x

**Published:** 2017-12-22

**Authors:** Gerusa Estelita Pires, Ana Carolina Peuker, Elisa Kern Castro

**Affiliations:** Programa de Pós-Graduação em Psicologia, Universidade do Vale do Rio dos Sinos, Avenida Unisinos, 950, prédio E01, sala 215, São Leopoldo, RS 93022-750 Brazil

**Keywords:** Hypertension, Perceived stress, Illness perception

## Abstract

The objective of this study was to describe and evaluate the effects of a pilot intervention on perceived stress, knowledge about hypertension, and illness perception among hypertensive and normotensive workers. The intervention consisted of two group sessions performed in the workplace aiming to reduce stress, increase knowledge about hypertension, and explore the effect on illness perception. The sessions included clinical aspects of systemic arterial hypertension, illness perception and health behavior, and strategies for stress management. Workers from a petrochemical industry (19 hypertensive and 14 normotensive) participated in the study by answering a biosociodemographic questionnaire, the Brief Illness Perception Questionnaire, the Perceived Stress Scale, and a quiz with questions about hypertension. The measurements were collected at an initial meeting to gather the participants and 90 days after the intervention. There was a significant reduction in the perceived stress levels of both groups, in addition to an increase in the perception of personal control and illness coherence. Normotensive workers also increased their knowledge about hypertension, while hypertensive patients increased the perception that treatment could control the illness. In conclusion, the pilot intervention generated positive effects and can be considered a strategy of illness prevention for normotensive workers and control for hypertensive patients.

**Electronic supplementary material:**

The online version of this article (10.1186/s41155-017-0080-x) contains supplementary material, which is available to authorized users.

## Background

Systemic arterial hypertension (SAH) is a chronic illness considered to be a serious global public health problem. It is one of the major risk factors for the development of cardiovascular disease, kidney failure, and stroke. In addition, it is a multifactorial clinical condition, attributed to factors such as heredity, age (aging), overweight, and lifestyle. The disease usually has no symptoms along its course, which makes many patients remain without diagnosis for an extended time. SAH is also a disease of difficult control, for even after diagnosis, due to the absence of symptoms, many people are unaware or do not follow the treatment and the necessary recommendations to control it (World Health Organization [WHO], 2013).

In this sense, WHO (2013) has sought to sensitize the private sector to the need to develop health programs, with attention to the prevention and treatment of hypertension and associated comorbidities. Furthermore, many businesses have recognized the importance of employee health to achieve core objectives. So, programs for health promotion and disease prevention may be seen as strategic initiatives to protect human and financial resources. By promoting risk factor reduction, businesses may avoid unnecessary health costs, enhance productivity, reduce absenteeism and turnover, and engage the employees through a demonstrated commitment to their well-being (Quintiliani & Sattelmair, 2007). However, there is a lack of health prevention interventions conducted in the workplace, especially in Brazil.

In addition to compliance with medical treatment, maintaining a healthy lifestyle is critical for the control of hypertension. Practicing physical activities, quitting smoking, decreasing alcohol consumption, following a balanced diet, especially with reduced salt consumption, and managing stress are recommended (American Heart Association [AHA], 2014; WHO, 2013). Stress is an important risk factor because elevated blood pressure (BP) may occur as part of an acute stress response (Steptoe & Kivimaki, 2013).

Stress occurs when individuals perceive and assess a situation as threatening to their well-being and exceeding their resources to face it (Lazarus & Folkman, 1984). The Transactional Stress Model supports that there are two processes acting as key mediators between events experienced by an individual and the perception that they are stressors: cognitive assessment (thought processes that sustain how events are interpreted and the cause attributed to them) and coping (efforts made by an individual to face a stressful event) (Wallbank & Robertson, 2013).

Knowledge, experiences, beliefs, and values can contribute to the way people assess situations and influence a person’s sense of competence and self-management in the face of events (Wallbank & Robertson, 2013). In this sense, the level of stress is associated with the perception of everyday events, including the way chronic diseases, such as SAH, are perceived. Thus, the perception that the illness is very threatening can generate stress and is also associated with other physical and psychological effects such as anxiety, fatigue, and depression (Hagger & Orbell, 2006; Israel, White, & Gervino, 2015; Westbrook, Maddocks, & Andersen, 2016).

The Common-Sense Model (CSM) postulates that when people think of an illness, people construct mental schemas that result from their reactions to internal and external stimuli (experiences, personality factors, etc.) (Leventhal, Meyer, & Nerenz, 1980). These schemas have a cognitive and an emotional representation and the content of these representations forms illness perception. From this perception, the individual defines the degree of threat to the health that an illness offers and the self-management strategies (related to the procedures for prevention and control and the action plans in which procedures are embedded), in order to cope with the illness (Cameron, 2003; Leventhal et al., 2001, Leventhal, Philips, & Burns, 2016a).

The content of illness perception is organized in identity (symptoms), causes (factors that originate the illness), chronic or acute timeline, cyclical timeline (course of illness), consequences (physical, psychological, and social impacts), personal control (how much one can control or achieve), and treatment control (effectiveness of treatment in control or cure) dimensions. In addition to these, the dimensions of coherence (illness understanding) and emotional representation (emotions associated with the illness, such as concern and fear) are also part of the model (Leventhal et al, 1980; Moss-Moris et al., 2002).

Interventions in clinical and health psychology using the CSM have shown positive effects on illness perception of patients with chronic diseases such as hypertension, as evidenced in a systematic review (Jones, Smith, & Llewellyn, 2015) that evaluated the effectiveness of nine interventions aimed at changing behaviors and beliefs about the illness. Participants were carriers of different chronic diseases, and post-test results indicated changes in participants’ health behaviors (lifestyle and return to work, for example). Two of the studies reported significant changes, with changes of perception about control, timeline, coherence, and dimensions of illness causal attribution. None of the interventions reviewed were performed in the workplace.

Another study (Petrie, Cameron, Ellis, Buick, & Weinman, 2002) relied on the CSM to examine if a brief hospital intervention, designed to alter 65 patients’ perceptions about their myocardial infarction (MI), would result in a better recovery and reduced disability. Patients were assessed in the hospital before and after the intervention and at 3 months after discharge from the hospital. The findings indicated significant positive changes in patients’ views of their MI and that they felt better prepared for leaving the hospital and subsequently return to work at a significantly faster rate than the control group. The patients in the intervention group also reported a significantly lower rate of angina symptoms than the control group after 3 months.

As for stress management interventions, a literature review (Varvogli & Darviri, 2011) identified key evidence-based techniques such as progressive muscle relaxation, autogenic training, biofeedback, diaphragmatic breathing, cognitive behavioral therapy, and mindfulness. These techniques were evidenced as effective for the design of interventions in health promotion for different publics and contexts. In the workplace, interventions for stress management with the use of cognitive, behavioral, relaxation, and/or meditation/mindfulness techniques are highlighted. The findings of these studies showed a reduction in participants’ stress levels, especially with interventions using combined techniques (e.g., behavioral relaxation and psychodynamic/cognitive behavior) (Bhui, Dinos, Stanfeld, & White, 2012; Limm et al., 2011).

In contrast, another study showed that only cognitive behavioral techniques produced significant effects (Richardson & Rothstein, 2008). Interventions for stress management can be an important tool for the improvement and maintenance of workers’ health, but it must be taken into account that the workplace is a very specific context in which there is little time to implement interventions, requiring proposals of brief and more dynamic interventions, in which the teaching material used must be more objective and with a modern layout, in order to attract and retain the participants during the intervention.

Regarding the CSM, no interventions were found for health promotion and illness prevention in the workplace under this perspective nor there has been any intervention with healthy groups under this model (Jones et al., 2015) even if studies already revealed the potential and relevance of the model also with healthy individuals. In the same direction, longitudinal studies are scarce, which makes it an emerging and promising area of study (Del Castillo, Godoy-Izquierdo, Vásquez, & Godoy, 2013; Quiceno & Vinaccia, 2010). As at the time of the design of the intervention and the selection of the participants of the present study, it was still unclear whether it would work or not, so it was decided to test the effects on both hypertensive and normotensive in order to better understand the phenomenon and subsequently improve the protocol according to each condition.

So, the pilot was to explore the potential of a single intervention to improve outcomes in both hypertensive and non-hypertensive population in occupational setting. In addition, in the face of the connection between hypertension and stress and the importance of this variable for illness control in hypertensive patients, stress management was included as the main outcome in the pilot intervention. Also, aiming to assess the level of satisfaction of the participants about the pilot, the acceptability was included as one of the outcomes during the intervention.

Therefore, this study aimed to describe and evaluate the effects of a pilot intervention on perceived stress, knowledge about the illness, and illness perception among hypertensive and normotensive workers. The general hypothesis was that the effects of the intervention would occur both in the hypertensive and in the normotensive groups. The specific hypotheses were (1) reduced stress levels, (2) increased knowledge about hypertension, and (3) general illness perception with significantly higher average adjust, being closer to the cutoff point of the B-IPQ instrument. Because it was a pilot and exploratory study, there was no clear hypothesis as to what dimensions of illness perception would change.

### Intervention protocol

The intervention protocol (Appendix) consisted of two weekly sessions, with and duration of 2 h each, in addition to an initial meeting used as a form of gathering the participants for the intervention. All the meetings were accompanied by the researcher. Table 1 shows the intervention description according to the TIDieR checklist (Hoffmann et al., 2014):

**Table 1:** Intervention Description and Replication (TIDieR) checklist

Item number	Item
Brief name	
1	Brief intervention for stress management and change in illness perception among hypertensive and normotensive workers: pilot study and protocol.
Why	
2	The study aimed to describe and evaluate the effects of a pilot intervention on illness perception, perceived stress and knowledge about the illness among hypertensive and normotensive workers. The main rationale was to improve the knowledge about hypertension, adjust the participants’ perceptions to the average threat level of the disease.
What	
3	The intervention group received a psychoeducation training program that took practitioners through clinical aspects of systemic arterial hypertension, life style and stress management, the methodology was expository, included group dynamics/exercises and classroom discussion. The participants received a folder, containing clarifications on the hypertension, a certificate of participation and a summary with tips for relaxation in stressful situations (available on https://osf.io/jyr6c/). Precise details of the components of the training program can be found in Appendix.
4	The detailed agenda can be found on Appendix.
Who provided	
5	The sessions were given by a psychologist, with a master degree in clinical psychology and a solid experience conducting group intervention for psychoeducation or change of health behavior.
How	
6	The sessions were presence, being held weekly and facilitated in groups of 4–8 participants, lasting 2 h each.
Where	
7	The participants were recruited at their workplace (petrochemical industry) and the sessions were carried out in the same location. The infrastructure included a private meeting room, chair, projector, computer (for slides presentation) and air conditioning.
When and how much	
8	The intervention was composed of two weekly sessions plus a first meeting called “Attraction”, in which the participants received information about how the intervention would work, gave their consent and responded the first stage of data collection. The first session lasted 1 h and the others 2 h. The sessions were set up at different times, according to the participants’ availability. Two groups occurred in the afternoon, from 2.00 p.m. to 4.00 p.m. (one of hypertensive and another of normotensive participants) and two groups in the morning, from 10.00 a.m. to 12.00 p.m., separated by condition (clinical or nonclinical).
Tailoring	
9	The intervention was not planned to be personalized, titrated, or adapted.
Modifications	
10	Intervention adherence or fidelity was not assessed.
How well	
11	There were no modifications during the intervention.
12	The mean (SD) number of intervention sessions attended was 2.05 for hypertensive and 2.29 for normotensive group (including the meeting of attraction). Sixteen participants completed the program—eight hypertensive and eight normotensive (48%). The reasons given for non-attendance to the intervention were a lot of work demand, work trip, or pre-scheduled medical procedure.

## Methods

### Design

The design is a quasi-experimental study, with pre and post-test measurements (Creswell, 2010).

### Participants

Thirty-three workers from a petrochemical industry (divided into two groups: 19 hypertensive and 14 normotensive), located in the greater Porto Alegre, Rio Grande do Sul, Brazil, participated. The aforementioned unit of the company had about 600 employees, and 422 worked during business hours (shifts between 7:00 a.m. and 5:00 p.m.), which was defined as viable for carrying out the research in the company. Of this total of day workers, 24 (6%) had previous diagnosis of SAH, according to a diagnosis made by the company’s cardiologist, through an annual health assessment. The evaluation was recorded in the health records of the workers and the results reported to the research team by the occupational health nurse.

Inclusion criteria for both groups were to be at least 18 years of age, to work in business hours, and to have at least complete elementary school. For the hypertensive group, it was also necessary to have a previous diagnosis of SAH. Initially, all patients with the illness (*N* = 24) showed interest in participating, and 19 (79%) attended the gathering meeting. The alleged motive for non-attendance of the others was time inconsistency between their professional activities and the intervention schedule.

For the normotensive group, all workers without hypertension diagnoses (*N* = 398) were invited. As exclusion criteria, it was established that normotensive individuals could not be carriers of chronic cardiac or kidney disease or have a personal history of stroke. Of this total, 22 (8.76%) expressed interest in participating, and 14 (3.52%) attended the attraction meeting and agreed to participate in the intervention. Of the total number of pre-test participants (*N* = 33), 16 (48%) completed the intervention (8 hypertensive and 8 normotensive), with absences/dropouts justified by travel or professional commitments at the time of the intervention. Participants who were unable to complete the sessions were contacted again with the possibility of resuming sessions in the next group meetings, with two participants returning and completing the intervention in subsequent groups. The criterion adopted was that the participant with an interest in completing the intervention should restart it from the first session, and it was not necessary to participate again in the gathering. The ages of the participants ranged from 39 to 62 years for hypertensive patients and 27 to 50 years for normotensive individuals. In addition, there were two female participants in the hypertensive group, while among the normotensive women, there were six women. This heterogeneity of sex and age, although increasing the ecological validity of the results, compromised the pairing of the two groups for later comparison.

**Fig. 1:**
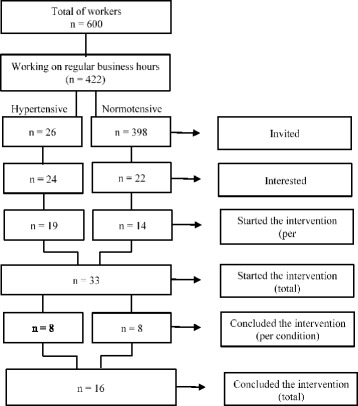
Total of participants per stage of the research

Figure 1 shows the participants by stage.

### Instruments

Biosociodemographic questionnaire: This included questions to collect personal data, such as age, sex, education, marital status, company time, in addition to the family history of SAH and a question on a scale of 0 to 10 for self-assessment about the current health status.

Brief Illness Perception Questionnaire (*Brief* IPQ: Broadbent, Petrie, Main, & Weinman, 2006) The Brief IPQ consists of nine items developed from the original instrument, the IPQ-R (Illness Perception Questionnaire—Revised). In the Brazilian version of the instrument (Nogueira, Seidl, & Troccoli, 2016), the subscale time dimension (item 8) was withdrawn because it obtained very low factor loads. And, in the present study, the item causal attribution and timeline were excluded. From the cutoff of 33 points, the illness is considered as threatening and, the larger the total, the greater the perceived threat (range of 0–70). The Brazilian version was also tested and validated with SAH patients. In the present study, the original instrument was adapted so that it could also be answered by healthy (normotensive) people.

Perceived Stress Scale (Cohen et al. 1983): Validated for Portuguese (Luft, Sanches, Mazo, & Andrade, 2007), it is composed of 14 items in a Likert scale of five points, ranging from “never” to “always.” The questions are divided into seven positive and seven negative ones and refer to the feelings and thoughts of the participants during the last 30 days. The total of the scale was obtained from the sum of the scores of the 14 items and the scores ranged from 0 to 56.

Health Quiz: Questionnaire prepared by the research group, containing 11 questions on the main clinical aspects of SAH, based on the dimensions of the CSM (Additional file 1). Participants should indicate whether each statement was “False” or “True.” Each question was worth one point.

### Data collection procedures

The contact with the company started from the work nurse, who was contacted for purposes of presenting the project. A meeting was held between the nurse and the members of the research group, in order to present the proposed research project for an intervention with hypertensive patients. Subsequently, the project was adjusted to include hypertensive and normotensive individuals, and the definition of data collection procedures occurred. After all adjustments, the letter of consent was signed by the Occupational Health Management.

Participants were invited to participate in the period from January to February 2016. They were sent an e-mail by the occupational health area of the company. The invitation was sent 2 weeks prior to the research. In the e-mail, the workers were invited to participate in the research and to know the proposal of an intervention for the control and prevention of hypertension. Data collection was divided into pre (gathering) and post-test (90 days after the gathering).

Three intervention groups were carried out, at different days and times, so that participants could attend them as they were available. The pre-test was performed at the end of gathering meeting, after the signing of the informed consent form. In the post-test, the same instruments used in the pre-test were filled out. The post-test collection was also performed in person and in a reserved place, occurring on two previously scheduled dates, according to the participants’ availability. The mean response time of the instruments was 20 min, and the questionnaires were applied by the researchers, with the help of a Psychology undergraduate student.

### Data analysis

The analyses were performed in the IBM SPSS (Statistical Package for the Social Sciences) program for Windows, version 22.0. Due to the distribution abnormality and sample size, non-parametric statistics were used. Descriptive analyses (means, standard deviation, frequencies, and percentages) were performed to know the results of psychological variables and biosociodemographic data. Inferential statistics were also used, with the Wilcoxon test to verify possible differences between pre- and post-test of the psychological variables of each group. Due to the heterogeneity of the sample, all comparisons between pre- and post-test were among participants of the same condition (hypertensive with hypertensive, for example). In addition, for the intra-group comparative analysis, the effect size measures were calculated by the Cohen method (Cohen, 1988), comparing the final with the initial means. This calculation makes possible the measurement of a potential real significance of the effect of the intervention in each group, by describing the size of the observed effect, which is independent of a possible misleading effect as a function of the sample size (Lindenau & Guimarães, 2012). For the interpretation of effect size (ES), Cohen proposed the following classification: insignificant (ES ≤ 0.19), small (0.20–0.49), medium (0.50–0.79), large (0.80–1.29), and very large (ES ≥ 1.30).

## Results

### Biosociodemographic data

The results presented refer to a sample of 33 participants, eight women (24%) and 25 men (75%), distributed between the two groups (hypertensive and normotensive). The mean age of hypertensive individuals was 48.95 (SD = 6.95) and of the normotensive individuals 36.21 (SD = 7.38). More than 60% of the participants had completed higher education/graduate school. The remaining 40% were divided into incomplete higher education/high school. In the hypertensive group, 84% had a family history of SAH or associated diseases, while 100% of normotensive individuals reported having a family history. Table 2 presents the main biosociodemographic data of the sample.

**Table 2:** Biosociodemographic data of the sample (*N* = 33)

Condition		Hypertensive		Normotensive	
		*N* (19)		*N* (14)	
		*M*	DP	*M*	DP
Mean age		48.95	6.95	36.21	7.38
Time of diagnosis of SAH^a^		2.68	5.82	–	–
Frequency/percentile		F	%	F	%
Sex	Female	2	10.5	6	42.9
	Male	17	89.5	8	57.1
Marital status	Separate/widowed	0	0	3	21.4
	Single	1	5.2	2	14.3
	Stable union/married	18	94.7	9	64.3
Time of work in the company	0 to 9 years	1	5.2	7	50
	10 to 19 years	5	26.3	5	35.7
	20 to 30 years	8	47.3	2	14.2
	Over 30 years	5	26.3	0	0
Education	Incomplete high school	1	5.2	0	0
	Complete high school	5	26.3	1	7.1
	Incomplete higher education	2	10.5	3	21.4
	Complete higher education	4	21	1	7.1
	Incomplete post-graduate education	7	36.8	8	57.1
	Complete post-graduate education	0	0	1	7.1
Takes medication for SAH	Yes	16	84.2	0	0
	No	3	15.8	0	0
Family history of SAH	Yes	16	84.2	14	100
	No	3	15.8	0	0

^a^Mean in years

*SD* standard deviation

### Acceptability of the intervention

At the end of each section, the participants completed an evaluation of the pilot intervention, answering the question “How would you classify the meeting in general?” (from 0 to 10, being unacceptable = 0 and acceptable = 10). The results showed that the mean overall level of acceptability for hypertensive group was 9.3 and for normotensive, it was 8.9.

### Pre- and post-test

#### Health Quiz

Normotensive individuals significantly increased the level of knowledge about hypertension in post-test (*p* = 0.01). However, there was no significant increase in the level of knowledge for hypertensive patients (*p* = 0.09). In any case, both groups already had good knowledge about the illness, according to pre-test evaluation. So, the specific hypothesis that both groups would increase the knowledge about SAH was partially supported.

### Psychological variables

#### Perceived stress and current health status

Perceived stress levels of both groups were significantly reduced in the post-test (hypertensive: *p* = 0.05, normotensive: *p* = 0.03). According to the effect size estimation, for the hypertensive group, a small effect (*d* = − 0.22; 95% IC − 0.25 to − 0.21) was detected on the perceived stress comparison. Regarding the results in the normotensive group, it was found a great effect in the perceived stress (*d* = − 0.65; 95% IC 0.686–0.630) indicating that there was a large reduction in the mean of post-test. These results support the specific hypothesis that both groups would have reduced stress levels.

Self-evaluation of the current health status of hypertensive patients increased significantly in the post-test (*p* = 0.01). In the pre-test stage, the mean of the evaluation was 6.89, while in the post-test, it was 7.56. Regarding the effect size, there was a representative effect in the current health status comparison (*d* = 0.79; 95% IC = 0.79–0.82), classified as large, indicating a representative increase of the mean in post-test. For the normotensive patients, there was no significant difference comparing pre and post of this variable (*M* = 7.86 pre; *M* = 8.23 post), the effect size also was unimpressive. Table 3 presents the pre- and post-test results and the effect size of perceived stress and the current health status.

**Table 3:** Perceived stress and current health status

	Hypertensive					Normotensive				
	Pre		Post		Sig	Pre		Post		Sig
	Mean	SD	Mean	SD		Mean	SD	Mean	SD	
Perceived stress	23.45	6.44	21.83	7.80	0.05	22.83	8.24	19.08	6.58	0.03
Health status	6.89	0.83	7.56	0.85	0.01	7.86	0.86	8.23	0.93	0.27
	Effect size (*d*)		IC_*d*_ 95%			Effect size (*d*)		IC_*d*_ 95%		
			Inferior		Superior			Inferior		Superior
Perceived stress	− 0.22		− 0.25		− 0.21	− 0.65		− 0.68		− 0.63
Current health status	0.79		0.79		0.82	− 0.01		− 0.00		0.03

*SD* standard deviation

### Illness perception

In the present study, the overall illness perception will be presented first and then the results of each item, according to the dimensions of the CSM. In the overall illness perception score, there was no significant difference for both groups compared to the previous score. Despite this, hypertensive patients already had a total perception below the threat level (pre = 27.8; post = 24.8). However, normotensive individuals maintained a perception that hypertension is slightly threatening (pre = 36.9, post = 35). Based on this, the specific hypothesis that both groups would have overall illness perception significantly closer to the cutoff point was not supported.

In the personal control dimension, both groups increased the perception that they could control or prevent hypertension (hypertensive: *p* = 0.02; normotensive: *p* = 0.00). In the treatment control dimension, both groups already had a high perception that the treatment is effective in controlling the disease; however, hypertensive individuals increased this notion significantly in the post-test (*p* = 0.05). In the coherence dimension, both groups increased the perception that they understood hypertension (hypertensive: *p* = 0.00; normotensive: *p* = 0.01) after the intervention. Regarding the consequences, identity, and emotional representation dimensions, there was no significant difference between the pre- and post-test in both groups.

According to the results obtained for the effect size estimates, comparing pre- and post-test, for the hypertensive group, the most representative effect it was found in the dimension of Coherence, being classified as large (*d* = 0.75, IC 95% 0.73–0.76), indicating an increase in the mean post-intervention score. In the treatment control (*d* = 0.56; IC 95% CI 0.54–0.58) and personal control (*d* = 0.55; IC 95% 0.53–0.57), medium effect sizes occurred. For the total mean score of Illness Perception, effect size was classified as small (*d* = − 0.38; 95% CI − 0.41 to − 0.36), indicating that the mean score was lower in post-test. In the other items, the estimates for the effect size were classified as small or insignificant.

In relation to the effect size in normotensive group, an effect classified as very large in the item coherence (*d* = 1.30; IC 95% 1.28–1.33) was detected, indicating a significant increase in the mean post-test score. Regarding the personal control item, a large effect (*d* = 1.02; IC 95% 1.01–1.03) was estimated, indicating that there was a large magnitude growth of the mean post-intervention. For total perception, the effect was again classified as medium (*d* = − 0.30, IC 95% − 0.33–0.27), indicating that there was a reduction of average magnitude on the mean post-test score. Regarding the other items, the effect size estimates were classified as small or insignificant. Table 4 shows pre and post-test results and the effect size of the illness perception dimensions.

**Table 4:** Illness perception

	Hypertensive					Normotensive				
	Pre		Post		Sig	Pre		Post		Sig
*Brief* IPQ dimensions/items	Mean	SD	Mean	SD		Mean	SD	Mean	SD	
Total perception	27.8	7.92	24.8	7.49	0.08	36.9	6.69	35	5.84	0.15
Identity	3.63	2.45	3.32	2.45	0.57	6.93	2,26	6.92	2.49	0.93
Consequences	4.26	2.42	4.11	2.92	0.69	8.79	1.52	8.85	1.21	0.86
Personal control	6.05	1.31	6.84	1.53	0.02	6.93	1.38	8.31	1.31	0.00
Treatment control	7.89	1.32	8.53	0.96	0.05	8.93	1.26	8.85	1.57	0.73
Emotional representation	5.12	2.72	5	2.56	0.86	6.39	2.78	6.9	2.36	0.75
Coherence	6	1.70	7.32	1.82	0.00	5.71	2.16	7.75	0.96	0.01
	Effect size (*d*)		IC_*d*_ 95%			Effect size (*d*)		IC_*d*_ 95%		
			Inferior		Superior			Inferior		Superior
Total perception	− 0.38		− 0.41		− 0.36	− 0.30		− 0.33		− 0.27
Identify	− 0.12		− 0.14		− 0.11	−0.00		− 0.02		0.01
Consequences	− 0.05		− 0.06		− 0.04	0.04		0.02		0.05
Personal control	0.55		0.53		0.57	1.02		1.01		1.03
Treatment control	0.56		0.54		0.58	−0.05		− 0.07		− 0.04
Emotional representation	− 0.04		− 0.06		− 0.02	0.19		0.17		0.22
Coherence	0.75		0.73		0.76	1.30		1.28		1.33

*SD* standard deviation

## Discussion

This study aimed to describe and evaluate the effects of a pilot intervention on perceived stress, knowledge about the illness, and illness perception among hypertensive and normotensive workers. Regarding stress, both groups showed a significant reduction in perceived stress levels, even though participants already presented moderate levels before the intervention. Preliminary support was found in a meta-analysis that showed that interventions with workers with high levels of stress were as effective as interventions with workers with low or moderate levels (Van der Klink, Blonk, Schene, & Van Dijk, 2001). This result reveals the intervention as a pertinent instrument for the management of stress in workers, being able to contribute as a strategy of prevention and health promotion in the organizational context.

Interventions to prevent illness and promote worker health are of great importance in the organizational context, owing to the great number of people that can be reached and the influence that companies have in promoting behavioral change of workers (Quintiliani & Sattelmair, 2007; WHO, 2013). In addition, people spend about 8 hours a day at work, which reinforces the need for health prevention actions in that context. However, there are many barriers to implementing interventions in the occupational context, such as the availability of staff time to participate in interventions during working hours due to workload. Another barrier is the difficulty of many companies in perceiving these programs as one of the best ways to prevent and promote health and, consequently, improve the organization’s results.

In the present study, the findings suggest that the intervention was acceptable to participants, which reveals the potential of the protocol for use in a larger trial. In addition, the adoption of mixed techniques such as psychoeducation and mindfulness may have contributed to stress reduction, as in the adoption of mixed techniques such as psychoeducation and mindfulness may have contributed to reduce stress, as in previous studies that have shown positive effects of multimodal interventions (Bhui et al., 2012; Limm et al., 2011; Ruotsalainen, Verbeek, Mariné, & Serra, 2015).

The fact that the overall illness perception score of both groups did not present any significant difference is supported by a longitudinal study in which was found that perception is not always as dynamic and able to be changed just by the course of the disease and its treatment, even with possible changes in knowledge about these factors (Castro, Kreling, Ponciano, Meneghetti, & Chem, 2012). Regarding the dimensions of illness perception, the significant increase in the perception of both groups about the dimension of personal control suggests that the participants strengthened their control beliefs and the notion of self-regulation. From a clinical point of view, this may be one of the most important dimensions for the control of hypertension, since both hypertensive and normotensive individuals, knowing more about their health and potential for managing hypertension, tend to adopt a more autonomous and less passive attitude towards risks.

The adoption of self-management strategies to control an illness requires that individuals not only perceive themselves as responsible for their health, but that they also see themselves as self-efficient to achieve the necessary change of lifestyle (Leventhal, Phillips, & Burns, 2016b; Tanenbaum et al., 2015). Self-efficacy has been linked to the perceived behavior control, as both concepts refer to the individual’s perceived ability to perform a behavior. Thus, perceived behavior denotes subjective degree of control over performance of the behavior itself, which should be viewed as an overarching construct that is comprised of two components: self-efficacy and controllability (Elie-Dit-Cosaque, Pallud, & Kalika, 2011; Manstead & Eekelen, 1998).

Considering that almost all the participants in the clinical group used medication for the control of hypertension, an increase in the perception about treatment control may represent an even greater contribution to this group. Among the dimensions of illness perception, this is the greatest predictor of adherence to treatment (Phillips, Leventhal, & Burns, 2016) and, as per the Necessity and Concerns Framework, patients who believe in the necessity of medication are more likely to be compliant (Foot, La Caze, Gujral, & Cottrell, 2016; Ross, Walker, & MacLeod, 2004). However, a previous study (Phillips, Leventhal, & Leventhal, 2013) revealed particularities of the role of beliefs for intentional and unintended adherence with hypertensive patients. Hypertensive patients who intentionally fail to follow treatment may be under direct influence of their common-sense experiences or beliefs, while unintended (spontaneous) adherence is related to the strength of the habit. In this sense, increasing the perception of treatment control may be of great relevance so that common-sense beliefs do not interfere with the intention of hypertensive individuals to adhere to the medication.

For the normotensive group, the strong belief about that treatment control may be related to the fact that all participants in this group reported having family members with hypertension. This was supported by a previous study which found that participants who had the experience of living currently or in the past with someone with hypertension had stronger beliefs on this dimension (Del Castillo et al., 2013). In this sense, while perhaps normotensive individuals are influenced by hypertensive relatives in their beliefs of control, a hypothesis is that having a strong belief that the treatment can control the illness, normotensive may be able to also influence hypertensive in their environment to use medications correctly and to adopt the therapeutic recommendations (e.g., lifestyle modification and stress management).With regard to the significant improvement in the perception of hypertensive patients about their health status after the intervention, this result may be associated with the increase of sense of personal control towards the illness, which may have contributed to these participants to feel as in better health status.

## Conclusions

The findings suggest that the participants of both groups may have felt more capable of managing stressful events from the understanding that stress levels do not depend exactly on situations, but on how participants perceive and face such events. Given this, the intervention may have contributed to the changing of the way participants manage stress in their daily lives and control their exercise in the management of hypertension. The findings also suggest that hypertensive and normotensive individuals are distinct groups, both in clinical condition and as regards beliefs about SAH. Anyway, the pilot intervention seems to have produced positive effects regarding the sense of self-management of both groups in face of the illness, especially from the increased perception about the personal control dimension. By increasing the sense of control and understanding (coherence dimension) on hypertension, participants may have felt more autonomous and empowered in the face of the illness, favoring the adoption of self-management behaviors and the perception of a better health status of hypertensive individuals.

Although this is a pilot study, its design was limited by small sample size, lack of randomization and control group. The reduced number of participants made it difficult to carry out additional inferential analyses, which would contribute to a deeper understanding of the phenomenon. In addition, because of the heterogeneity of the hypertensive and normotensive groups, pairing was not possible. As perception, in the identity dimension, has remained high for normotensive individuals and hypertensive individuals have maintained a general perception below the level of threat, it is suggested that the intervention be personalized (for example carry out the intervention with greater focus on the dimensions that need adjustment) in later studies, being carried out in separated groups, in order to meet the needs of each condition. In this sense, a previous analysis of the pre-test measures and a qualitative mapping may be important to identify which items require customization for each group. Also, in terms of feasibility and acceptability of the pilot by the participants, the study was limited by the fact that a single item was used to explore it, might a richer understanding would have come from a qualitative interview.

Future research may replicate the intervention protocol with larger samples and in different work contexts. It is also recommended that a reward system, based on contingency management, be used to encourage adherence to sessions and collections, in order to increase the positive effects of the intervention. Another recommendation is that additional relevant measures be included, such as the application of self-efficacy scales, aiming at the analysis of associations with illness perception, as well as biological measures. Additionally, in a larger intervention study, a process analysis could be undertaken to identify how the intervention might have worked. The CSM and the Transactional Stress Model were valid to increase the participants’ health sense of empowerment and self-regulation.

New interventions may associate them with other models (such as the Necessity and Concerns Framework) with the aim of maximizing the effects of the intervention, including the creation of action plans aimed at changing behaviors, especially for hypertensive individuals. Also, it would be necessary to longitudinally assess the illness perception and stress and to include measures of behavior change, first to identify if the changes in perception and stress would be sustained and second if the changes in perception would have an effect on the adoption of healthy behaviors. In future studies also, it is essential to take into account that the organizational context implies a different pace of research, with a greater sense of urgency and that it needs constant adaptation to the many uncontrollable variables that permeate the conduct of research in a company.

The pilot group intervention seemed to be relevant to improve knowledge about the illness and the sense of self-management of the participants. In addition, the interaction between the participants facilitated exchanges of experiences and may also have favored the positive results found. Although it was a pilot study, the findings reinforce the importance of clinical psychology for the development and application of health education interventions as strategies for illness prevention and control in the workplace. This study is unprecedented in bringing clinical and health psychology to the organizational context. In this perspective, the study contributed to a greater knowledge of the psychological and behavioral aspects associated with hypertension and for the development of a pilot intervention protocol. The results of this study may be helpful in subsidizing future interventions aimed at the stress management and prevention and control of hypertension, especially in the organizational context.

### Additional file

Additional file 1: Health Quiz. (DOCX 28 kb)

## Appendix

Description of the intervention protocol and the schedule of intervention meetings:

**Table 5:** Description of the intervention protocol

Meeting	Objective	Procedures
Attraction	Introduce the project proposal and sensitize workers, arousing interest in joining the intervention.	Introduction to the Project, the stages of the intervention and the study. Sensitization to the theme “psychological aspects and behavior in health” and to the idea that the participants could become multipliers of the knowledge acquired, with their relatives and other people close to them. Pre-test: Clarification of doubts and data collection.
Session I: Perception and clinical aspects of SAH	To raise awareness among the participants about the influence of psychological aspects on health behavior and to increase their knowledge about hypertension, promoting greater self-management in relation to the illness.	Introductions: introduction of the facilitator and definition of psychological agreement with the group (secrecy, respect, schedules, participation). Participants introduced themselves by informing name, work area and what hypertension represented for each one. Reflection on perception: it was proposed that participants should close their eyes, breathing deeply and thinking about the following question: “What does SAH represent (as I perceive it) to me, does it draw me closer to or distract me from well-being?”. After about 10 s, participants were asked to open their eyes and write down what they thought, keeping the note to themselves. Then they were asked how they felt thinking about it (the idea was not to read what they wrote, but to have someone comment on what they felt if they wanted to share). At that moment, there was a connection between perception and hypertension, highlighting the importance of how participants perceived hypertension for the adoption of illness prevention and control behaviors. Cognition and behavior—Perception and Hypertension: exposition of the main components of perception, as well as cognition and behavior (thought, feeling, behavior), including the use of metaphors and everyday examples, to emphasize the influence of perceptions/beliefs on behavior in health. Examples of metaphors: The image of a ferocious lion was used, associating it with the fact that some people perceive SAH as very threatening, while others may perceive it as a cat’s cub (images of a lion and a cat were presented). When, in fact, what is appropriate is that it is neither of these extremes, but rather that hypertension is seen as a serious disease, but capable of total prevention and control. The image of the scales was also used in order to illustrate that the ideal is not to weigh the scales very much to one side or another, but rather that the scales be balanced. Health Quiz (individual): activity to assess the level of the participants’ prior knowledge about SAH. Participants received a questionnaire with affirmations to mark true or false. The questions addressed the content of the illness perception dimensions, the Common-Sense Model (CSM). They were asked to answer individually, remaining with the questionnaire until the end of the next activity. It was reinforced that they would not need to share their answers with the group and that if they did not know how to answer a question; the doubts would be clarified in the group activity without exposing the individual sheets. Health Quiz (collective): Psychoeducational activity on hypertension. After answering individually, the participants answered the same questions, discussing the correct answers with the facilitator and other participants. At the end, the individual questionnaires were collected, with the purpose of comparing the responses of the first application (pre-test) with the application of the same questionnaire (post-test) to be performed in the last session of the intervention. Closing
Session II: Stress management	Psychoeducation participants on stress and management strategies, improving their resources for everyday stress management	Review of the previous session: participants were asked to share what they scored in relation to the previous session. With this information, the facilitator connected the first session with the current one. Psychoeducation about stress: brief presentation of the risk factors for hypertension, focusing on the stress factor and the relationship between stress and illness. Presentation of the concept of stress (Stress Transactional Model), highlighting the individual as active in the stress management process, from the way everyday events are perceived to the strategies adopted to deal with stressors. Experiential technique “Thoughts and emotions”: a volunteer from the group was asked to share an unpleasant situation that has happened to him/her in recent weeks, verbalizing the feelings, emotions that he/she experienced during the situation. Then the person was asked to stand in the middle of the circle formed by the group and that the large group interpreted the thoughts and emotions described by the person, repeating verbally several times. In the activity processing, both the group and the volunteer were questioned about how they felt during the activity. It was exposed to the group that besides the initial suffering we have secondary suffering, generated from the way we perceive and react to stress, which can cause even more suffering, depending on how the individual handles the emotions generated by the situation. The importance of perceiving and reacting positively to a more effective coping of situations was highlighted. Mindfulness-based stress reduction technique: The technique was presented as a resource to be used in unpleasant moments in order to assist in stress management. The technique is structured as follows: *stop* (step one): Choose to exit the autopilot by bringing conscious attention to the present moment. After that, pay *attention* (step two) to what is happening at the moment in your body, your emotions and thoughts. Direct your attention and focus simply on *breathing* (step three). *Enlarge* (step four) your consciousness again, including the perception of the body as a whole and the situation you are experiencing. Notice now that you can *respond* with awareness right now. Participants received a piece of paper with the description of the technique and were informed that this exercise could be used in any situation and that, like any practice, its regular cultivation, even in neutral situations, potentiates the positive result in difficult moments. Mindfulness Initial Practice (Mindfulness-based stress reduction): Participants were asked to sit comfortably on the chair, keeping their eyes open or closed. From this, they were led step by step for 5 min to be attentive to their breathing, to let their thoughts flow without judging whether they were good or bad, trying to experience the present moment. This technique was used to demonstrate how full consciousness training can improve self-perception and reflect on the body, mind, and experience as a whole, further contributing to the choice of behaviors that are in accordance with what is important in each one’s life. Video “The unwelcome guest”: presentation of the video, which addresses perception and behavior in the face of unpleasant situations, moments of anger. Processing: discussion of the impressions of the participants about the video. It has been argued that people can stop doing things that are important to themselves by being stuck with negative thoughts and emotions generated in unpleasant moments. In this sense, the active role of the individual to deal with stress was worked out; from the way he or she perceives and coping stressful situations. “Values” technique: From the discussion of the video, the participants were invited to think and write down a life value, which they think they can connect to contribute to the improvement of their health. It was then opened up for discussion about what the participants chose and the importance of connecting to their values so that they could change some behavior. Folder: delivery and presentation of the folder about hypertension. Closing and evaluation: Final thoughts on the meeting and participants expressed their impressions and what they considered important for themselves. Filling out of questionnaires and delivery of certificates to participants.

**Table 6:** Schedule of the intervention meetings

	Duration	Activity	Variables	Instruments
Attraction	10 min	Introduction to the project and the research group	–	Biosociodemographic questionnaire, Illness perception scale, Perceived stress scale
	10 min	Sensitization about “psychological aspects and health behaviors”		
	10 min	Introduction to the stages of the intervention and the study		
	30 min	Clarification of doubts and initial data collection (pre-test)		
Total:	1 h			
Session 1	5 min	Introduction and agreement	Illness Perception, knowledge	Pre-test of the Health Quiz and satisfaction assessment
	20 min	Introductions of participants		
	10 min	Reflection about perception		
	30 min	Cognition and behavior Psychoeducation		
	10 min	Health Quiz (individual—pre-test)		
	30 min	Health Quiz (collective)		
	15 min	Closing and evaluation		
Total:	2 h			
Session 2	10 min	Review of previous session	Illness Perception and perceived stress	Post-test of the Health Quiz and satisfaction assessment
	30 min	Psychoeducation about stress: risk factors, perception and stress		
	15 min	Experience “Thoughts and emotions”		
	10 min	P.A.R.A.R (STOP) technique and initial practice of mindfulness		
	20 min	Video “The unwelcome guest”		
	15 min	Technique “Values”		
	5 min	Folder		
	15 min	Closing, evaluation and delivery of certificates		
Total:	2 h			
