# Supplementary material for: Brief intervention for stress management and change in illness perception among hypertensive and normotensive workers: pilot study and protocol
Source: Psicol Reflex Crit. 2017 Dec 22;30:26. doi: 10.1186/s41155-017-0080-x (PMC6974342; doi:10.1186/s41155-017-0080-x)
Supplement: Supplementary file 1 — Health Quiz. (DOCX 28 kb) [file 41155_2017_80_MOESM1_ESM.docx]

**Additional file 1**

**Health Quiz**

**1. Hypertensive should have Hypertension care only when I feel sudden headache**

( ) True ( ) False

**2. Hypertension is incurable**

( ) True ( ) False

**3. Having Knowledge about hypertension and its care is important to control the disease**

( ) True ( ) False

**4. The main factor for hypertension is heredity**

( ) True ( ) False

**5. When hypertension is untreated it can cause other diseases**

( ) True ( ) False

**6. Hypertensive should initiate the treatment when the blood pressure is high**

( ) True ( ) False

**7. To control hypertension, it is only necessary to take the medicine recommended by the doctor**

( ) True ( ) False

**8. The sedentary lifestyle does not represent a threat for the prevention of Hypertension**

( ) True ( ) False

**9. In the diet, salt is the great enemy of hypertension**

( ) True ( ) False

**10. A diet rich in fruits and vegetables can impair the control of hypertension**

( ) True ( ) False

**11. My feelings can influence on the elevation of blood pressure**

( ) True ( ) False
